# Changing the Culture: Improving Helmet Utilization to Prevent Traumatic Brain Injury

**Published:** 2020-07-16

**Authors:** Brandon Lucke-Wold, K Pierre, F Dawoud, M Guttierez

**Affiliations:** 1 Department of Neurosurgery, University of Florida, Gainesville, Florida, USA; 2 School of Medicine, University of Florida, Gainesville, Florida, USA; 3 School of Public Health, East Tennessee State University, Johnson City, Tennessee, USA; 4 California Department of Publich Health, CDC Public Health Associate, Richmond, California, USA

**Keywords:** Helmet initiative, Traumatic brain injury, Safety, Community champions

## Abstract

**Background:**

Several groups have instituted helmet initiatives with varying success across the world. Helmet use has been well documented to prevent traumatic brain injury. Despite the known benefits, many people, including university students, refuse to utilize helmets when riding bikes, mopeds, or motorcycles. We recognized a need within our community regarding the lack of helmet use at University of Florida and developed a program to institute change.

**Methodology:**

We identified community champions and hosted weekly round table discussion initiatives. Through these round table discussions we identified events already going on within the community and developed new opportunities to promote helmet use. We had stories from survivors and parents, utilized school administration support, and partnered with local bike shops.

**Results:**

The pilot initiative was successful in increasing awareness across the city and got stakeholders excited in the process. It also spearheaded more data driven initiatives that will look at reduction of traumatic brain injuries in the clinical setting.

**Conclusion:**

This project highlights the University of Florida Helmet Initiative that has already generated renewed interest in safety and traumatic brain injury prevention. The school of nursing has implemented safety protocols and further support is being garnered by the administration across campus. Most importantly we have identified community champions that will carry the work forward.

## Background: Review of Helmet Initiatives

Various helmet initiatives and campaigns have been tried throughout the world (Table 1) and have generally been successful in their goal of increasing helmet use and decreasing brain injury. A common objective in many of the strategies used is making helmet wearing a norm, considering that many people do not wear helmets because their peers do not [1]. Efforts toward this goal were made through competition, events, and safety-orientated curricular and extracurricular activities. As an example, the Stanford transportation department started a “Bike Safety Dorm Challenge” which quickly lead to many people wearing helmets [2]. Another common reason for not wearing helmets is lack of knowledge. Therefore, another major goal was increasing awareness and knowledge about helmet use and prevention of head injury using videos, presentations, testimonies, and parent and teacher education. Lastly, helmet expense was a significant barrier [1]. Therefore, many of the initiatives and campaigns worked to decrease the cost of helmet use through programs or discount coupons. Some also provided free helmets. As an example, in the Trauma Nurses Talk Tough initiative, this cost issue was not addressed, which may explain why there was no significant increased use in helmets though there was a significant increase in students who reported that they would use helmets if they had them [3].

Funding for the projects varied. It generally came from organizations that aim to improve societal health outcomes. For example, the Cambodia helmet initiative was funded by the Centers for Disease Control (CDC) and the International Union for Health Promotion and Education (IUHPE) [4]. The Seattle Children’s Bicycle Helmet Campaign was funded by the CDC and the Snell Memorial Foundation [5]. The CDC also sponsored the Uganda Helmet Vaccine Initiative [6]. The Stanford Bike Safety Dorm Challenge, a more local initiative, was funded by the Parking & Transportation Services department of the university [13].

Many of the initiatives and campaigns assessed the outcome of their efforts. Methods to assess helmet usage included surveys and direct observation. For example, the school-based intervention in Cambodia involved roadside observations of the number of students who wore helmets before and after the intervention [4]. Hospital-based interventions generally would assess whether there was a significant reduction in the number of brain injuries after the implementation of the initiatives and campaigns. One hospital-based initiative found that hospital casualty rates halved for children after the implementation of a campaign [7].

Of note, the legislation of mandatory helmet use has been implemented in the past. Although these laws tend to be controversial, may contain ethical issues, and are likely to be more difficult to implement due to pushback, they have generally been successful in increasing helmet use and reducing injuries [8,9].

## Ideal Helmet Initiative Implementation/Design

Implementing an ideal helmet safety initiative, much like any other public health initiative, relies on multiple key factors becoming integrated collectively to achieve an efficient, impactful, and self-sustaining intervention. Features that define such successful initiative include an evidence-based intervention model, effective community engagement, partnership with key stakeholders/legislators, plan for sustainability, and defined criteria for evaluation of the intervention [10–16]. However, identifying the ideal intervention can be complex because there exists an array of evidence-based interventions. The multiple targets of these various interventions further complicate this matter. In order to effectively identify the ideal intervention and corresponding target, an assessment of the community needs and assets must be properly conducted.

An assessment of the community is a critical first step in designing and implementing a helmet initiative [16]. This is critical in order to define the area of disparity and which target will most effectively benefit as a result of addressing this disparity. Some of the key contributing factors that result in low helmet use include lack of public awareness of helmet benefit, lack of access to helmets, low socio-economic status, lack of perceived susceptibility, and lack of cultural subjective norm [4,14,17,18]. Any one or a combination of these may be the key cause of low helmet use. As a result, developing an ideal intervention must be tailored to address all or most of these key issues directly and effectively. One model that has been particularly effective in predicting behavioral change and interventional success is the Health Belief Model (HBM) [16,19,20]. Studies based on HBM have found that those who have increased perceived benefit/susceptibility, increased cues to action, and decreased barriers were the most likely to adopt using a helmet regularly [19,20].

In order to implement an effective plan that is sustainable, partnering with community stakeholders is essential [16,18]. Stakeholders can be a vital source of funding, community knowledge, and supporting assets. These stakeholders can range from political office holders to local community business owners and can aid in the development of a more effective initiative by assisting in identifying targets and defining the scope of the intervention [16,18]. Stakeholders may themselves be assets by gaining direct access to the community. Effective community engagement is a defining feature of a successful helmet initiative and thus being able to gain access to the public is paramount. This can be achieved through marketing campaigns that are aimed at the intervention target or direct access through community groups such as schools or faith-based organizations [18]. Several extremely successful interventions partnered with local media groups in order to spread public awareness [4,6,14,17,18]. The key to effective marketing is through a “simple, consistent, and memorable” message that is socially and culturally appropriate to the target audience [18]. Initiatives with wide scope that can reach multiple targets in the community can be extremely impactful, however the key to efficiency is in directly reaching the target audience effectively.

Funding for a helmet initiative is equally essential. Not only for initially implementing the initiative, but also for long term sustainability in order to achieve lasting results. This concept works hand in hand with both partnership and community engagement. Helmet initiatives that were able to integrate key stakeholders of the community for financial support were more capable of achieving a self-sustaining model [4,14,16,18]. The World Health Organization (WHO) outlines three strategies for funding helmet initiatives; first through sponsorship by corporate and community business leaders with common goals, second through partnership with charitable organizations which can help supply funding, and finally through re-investment of funds directly generated by the initiative [18]. Helmet use initiatives with a legislation aspect have used non-compliance fines, helmet sales, or motorcycle registration fees to re-invest in maintaining the initiative itself [4,14,18].

Finally, a mechanism of monitoring of the initiative goals is necessary for evaluation of effectiveness and continued growth overtime [16,18]. There are multiple indicators that can be assessed to measure initiative effectiveness depending on the initiative goals. These can range from helmet use rates to public awareness rates. These must be defined early in initiative development and assessed at regular intervals [18]. Data from these self-monitoring mechanisms can inform areas of improvement that then can be used to re-shape the initiative to meet its primary goals and scope [16,18–20]. Integrating this along all levels of initiative implementation can help form a long-lasting initiative that is flexible and efficient.

## Implementation at University of Florida

During orientation week for incoming residents and nursing staff, a presentation was given regarding quality improvement initiatives. During this session, new employees were encouraged to bring forward current problems in the community. It was noted that the majority of students and citizens in Gainesville do not utilize helmets during their commutes. This was in stark contrast to other areas of the country where new employees had previously lived. Thus the helmet initiative project was born. The health champions including residents and public health students met to design a plan. The first step was to determine the depth of the problem. With the assistance of volunteers, including health sciences students, documented tallies throughout campus showed that ~11% of individuals utilized helmets. Roughly 1000 bikers, motorcycle riders, or moped riders were observed.

After confirming that a problem exists, a multi-disciplinary team was developed including clinicians, nurses, public health students, and community champions. The community champions included local police officers, a mother who had lost her teenage child due to not wearing a helmet during an accident, bike shop owners, and community health organizations promoting community safety.

After the stakeholders were identified, the neuromedicine interdisciplinary clinical and academic program sponsored monthly meetings to discuss how to implement helmet use and safety. Although there was a limited budget, food was sponsored at these events. Several ideas were brainstormed including getting student athletes to wear specially designed helmets, videos regarding collisions without helmets during orientation week that would include interviews with survivors and/or parents of the deceased, and free bike helmets. Fortunately, the bike helmet option was feasible, which was not the case for several prior published studies. A prior grant had secured helmets to be distributed to students. The students had to document use and fill out certificate of need. We began to implement this quickly to get helmets to students. We also hosted events at local bike shops and community centers to encourage participation.

With several rounds of iterative feedback, it was decided that changes in legislation would be most effective going forward. Starting small we reached out to Deans in the respective health sciences campuses. The School of Nursing was responsive and implemented a helmet policy for all of their students on campus. The policy included disciplinary action if a student was caught not wearing a helmet. We are hopeful to eventually implement this policy across campus once more data is gathered from the nursing pilot project.

From the clinical standpoint, we are collecting further data regarding the patients admitted following motorcycle, moped, and bicycle collisions that were unhelmeted. We plan to mark initial hospital admissions and severity of injury prior to the initiative and again post-initiative. Our proposed hypothesis is that by increasing helmet use by at least 10%, there will be clinically significant reduction in severe traumatic brain injury. The use of continued community champions is critical in keeping the momentum going.

## Summary

Helmet initiatives have been shown to be safe, effective, and clinically beneficial. Utilizing experience from past initiatives, we are piloting a multi-disciplinary public health outreach endeavor to promote helmet use. We have identified public health champions, encouraged initial successes, and developed a plan to clinically measure meaningful change. In subsequent papers, we hope to further lay groundwork for how these projects can be implemented at other centers while providing data regarding the clinically observed changes in traumatic brain injury metrics at University of Florida.

## Figures and Tables

**Table 1:** Helmet initiatives and campaigns.

Initiative/campaign	Country	Setting/Target population	Intervention(s)	Outcome
Trauma Nurses Talk Tough [3]	United States	Students in 6^th^–8^th^ grade. & Students in 9^th^–12^th^ grade.	Presentation by trauma nurses about cases of people who have been injured. Note: students were not provided with helmets.	Significant increase in 6^th^-8^th^ graders (n=372) who answered yes to, “If you have a helmet, would you wear it.” No significant increase in the use of helmets
Pense Bem-Caxias do Sul Project [10]	Brazil	High-school students.	Video portraying an injured young victim Subsequent lecture on trauma prevention.	Significant increase in the use of helmets (n=1049)
ThinkFirst for Teens injury-prevention program [11]	United States	14–15-year old high-school students.	Video featuring testimony from victims of trauma Subsequent lecture by trauma nurse on trauma prevention.	Improved self-reported helmet compliance. (n=177 for pre-program survey; n=191 for post-program survey)
Uganda Helmet Vaccine Initiative [6]	Uganda	Motorcycle riders in the general population.	Radio messages addressing helmet use. Motorcycle operator workshops where participants received free helmets.	-
Helmets for Kids [4]	Cambodia	Students in primary school who used bicycles or motorcycles as a form of transportation.	Students were provided with free helmets. Students and teachers were educated on traffic safety. Parents were asked to encourage their children to wear helmets. Curricular and extracurricular activities regarding helmet use were implemented at the schools. TV commercials, billboards and radio ads promoted helmet use.	Significant increase in the use of helmets (0.46% to 87.9% of students; n=9752)
The Seattle Children’s Bicycle Helmet Campaign [5]	United States	School-aged children from 5–14 years of age.	A multifactorial approach including “stories in the print and electronic media, public service announcements, press conferences, posters, brochures, stickers, health fairs, bike rodeos, school and youth programs, and a discount coupon.”	Increase in helmet-use from 5.5% to 40.2% over 5 years. Reduction of bicycle head injuries by ~67% (n=701)
Stanford Bike Safety Dorm Challenge [12]	United States	Undergraduate students.	A dormitory competition- student dorms with the highest percentage of residents that have signed a pledge to wear a bike helmet and to follow the rules of the road win a trip to a nearby lake. Various discount programs.	951 students participated in this challenge in 2012 [13]
Colorado Community Bicycle Helmet Campaign [14]	United States	8600 elementary school students and their families.	Discount programs for low-income families Educational presentation at local schools and community events. Media promotion Physician education	Increase of helmet use from 9.9% to 37.1% in 2 years
Hospital-led Bicycle Helmet Wearing Promotion Campaign [7]	United Kingdom	5–15-year-old residents of Reading, West Berkshire	A hospital led community program involving: “School-based talks Age-specific information True case scenarios/videos of head-injured children A demonstration using an egg and small helmet to illustrate the effect of a head injury with and without a helmet information on how to wear a helmet properly a low-cost helmet purchase scheme”	Increase in self-reported use of helmets from 11% to 31% five years later Decrease in hospital casualty rates in children under 16 from 112.5/100,000 to 60.8/100,000 Decrease in head injury as a percentage of bicycle injury from 21.6% to 11.85%
Bicycle Helmet Promotion Among Low-Income Preschool Children [15]	United States	Low-income preschool students.	“ Classroom activities with children Education of parents during school meetings and home visits Fitting and distribution of helmets A bicycle skills and safety ‘rodeo’ event Requiring children to wear helmets while riding on school grounds”	Increase in observed helmet usage from 43% to 89%
